# Workplace precrastination: conceptualization and scale development

**DOI:** 10.3389/fpsyg.2025.1679477

**Published:** 2025-10-06

**Authors:** Jie Guo, Inchul Cho, Sushil Nifadkar

**Affiliations:** ^1^Management and Information System Department, University of North Georgia, Oakwood, GA, United States; ^2^Management and Information System Department, University of North Georgia, Dahlonega, GA, United States

**Keywords:** precrastination, procrastination, scale development, motivation, performance, attitude

## Abstract

Workplace precrastination is the phenomenon wherein individuals tend to rush task-related activities at work and complete them as soon as possible. Due to its prevalence in everyday life (e.g., answering emails quickly, finishing work rapidly), precrastination has received increasing media and scholarly attention. However, despite growing interest in the topic, no psychometrically sound measure exists to capture workplace precrastination in organizations. We define workplace precrastination as the tendency of employees to complete an official task sooner than the expected deadline. This tendency manifests in three distinct dimensions: (1) immediate start, (2) rapid progress, and (3) early completion. Using three samples and a multi-wave approach to data collection, we develop a new scale to measure workplace precrastination and test its construct validity. We also examine whether precrastination is conceptually and empirically distinct from procrastination. Our findings suggest that workplace precrastination is distinct from procrastination and that it contributes unique variance to several organizationally relevant behavioral and attitudinal constructs.

## 1 Introduction

*Speed is the essence of war. – Sun Tzu (The Art of War).*

*Do not wait. The time will never be just right. – Napoleon Hill.*

Precrastination is a prevalent workplace phenomenon, particularly in environments where organizations operate under strict deadlines. External competition and frequent shifts in organizational priorities force employees to respond rapidly to emerging challenges (Shipp and Richardson, 2021). Indeed, organizations often expect employees to complete their tasks well before the deadlines, and even reward them when they do so (Batt and Terwiesch, 2017; Indeed, 2024). Thus, it is not surprising that managers often perceive employees who start work activities early as conscientious and high performers (Yam et al., 2014). However, while hastening can produce positive short-term results, undue rush to complete tasks can also have negative consequences such as disabling injuries, high levels of stress, poor decision-making, and decreased quality (Berry and Tucker, 2017).

With the prevalence and potentially severe consequences of rushing to complete tasks, scholars have recently started to study this phenomenon in lab settings. Rosenbaum et al. (2014) first coined the term precrastination to examine people’s propensity to initiate task-related activities quickly and complete them as quickly as possible even at the expense of extra physical effort. Subsequent lab studies have reported that precrastination is prevalent in several other domains, such as memory retrieval (VonderHaar et al., 2019), animal decision-making (Wasserman, 2019), and task sequencing (Fournier et al., 2019). Precrastination also influences economic decision-making (O’Donoghue and Rabin, 1999) and consumer behavior (Zhu et al., 2018). Indeed, precrastination can be seen in everyday life in behaviors such as replying to emails too quickly, submitting reports well before the due date, and making hasty business decisions. Because of preponderance of precrastination in our daily lives, it has garnered considerable media attention as well (e.g., DeMelo, 2019; Richtel, 2014).

Given the significant influence of precrastination on various aspects of workplace behavior, there is an urgent need to study it systematically within organizational contexts. However, several challenges arise in this regard. First, the ecological relevance of prior research to the workplace is limited as precrastination has predominantly been studied in laboratory settings using undergraduate samples (Fournier et al., 2019; Rosenbaum et al., 2014; VonderHaar et al., 2019). This raises concerns about the external validity of these findings (Mitchell, 2012). Second, the commonly cited definition, “hastening of subgoal completion, even at the expense of extra physical effort” (Rosenbaum et al., 2014, p. 229), may not be well-suited to modern work environments where physical exertion is rarely a central concern (Tepper, 2007). Further, the costs or benefits associated with goal completion in organizations are often performance-based rather than physical-based exertion. Third, as the conceptualization of precrastination has evolved, it is essential to integrate these developments in an appropriate manner. Initially, precrastination was defined as hasty task *completion*, or at least *initiation* (Rosenbaum et al., 2014), and a sense of urgency to make *progress* was later included (Rosenbaum and Sauerberger, 2019).

In the present study, we aim to address the above three concerns regarding the current literature on precrastination. First, we conduct a field study using samples of working employees to address the external validity concerns stemming from the large number of lab samples in the past. Second, we recognize that the modern workplace often does not involve extensive physical labor, which was a main focus in previous precrastination studies. Therefore, we measure precrastination in the workplace and develop new items specifically suited for modern office employees. Third, as precrastination researchers have refined the definition of precrastination over the years, we integrate their evolving views to better capture the multidimensionality of workplace precrastination. Specifically, to comprehensively incorporate the unique and temporal aspects of precrastination, we propose that workplace precrastination tendency can manifest at different stages of task completion such as immediate start, rapid progress, and early completion of official tasks.

In light of the above, the purpose of this study is to extend the body of existing research on precrastination by offering a clear conceptual and operational definition of *workplace precrastination*. We begin by reviewing relevant research across disciplines and then develop and validate a multidimensional measure that captures workplace precrastination across three temporal phases. We establish the newly developed scale’s discriminant and incremental validity in relation to workplace constructs, including procrastination. Finally, we examine key antecedents and outcomes to clarify the construct’s role in organizational settings. This work provides a theoretically grounded, empirically validated tool to advance future research on workplace precrastination.

### 1.1 A brief literature review on precrastination

Why do some people precrastinate, that is, complete tasks quickly and long before deadlines? Cognitive psychologists suggest that precrastination serves to reduce cognitive burden, as unfinished tasks can be mentally taxing (VonderHaar et al., 2019). In behavioral economics, precrastination (termed preproperation) reflects a tendency to act prematurely to gain immediate psychological rewards, even when waiting would yield better outcomes (O’Donoghue and Rabin, 1999). Consumer research links it to “the mere urgency effect,” where individuals respond to spurious urgency, such as the illusion of an impending deadline (Zhu et al., 2018). During the COVID-19 crisis, social psychologists invoked the concept to caution against resuming normal activities prematurely, as such haste may not be the most prudent approach (Rosenbaum, 2020).

Although precrastination has been investigated in lab settings (Rosenbaum and Sauerberger, 2019), its conceptualization and measurement face at least four critical issues. First, studies on precrastination have primarily relied on observable behaviors in tasks such as carrying buckets or transferring objects in lab settings (Fournier et al., 2019; VonderHaar et al., 2019), which offers limited external validity, especially in organizational settings, and discounts the influence of social contexts (e.g., Berkowitz and Donnerstein, 1982; Mitchell, 2012). Anecdotal evidence suggests that precrastination manifests in more complex ways in organizational life, often impacting official tasks and overall productivity (Berry and Tucker, 2017). Thus, without examining precrastination within the broader organizational contexts, scholars may overlook its critical influence on employee behaviors and performance.

Second, although precrastination has recently gained attention, it is often mistakenly treated as merely the absence or low level of procrastination (Blinch and DeWinne, 2019; Rosenbaum et al., 2014). We argue that procrastination and precrastination are distinct constructs. In their theoretical work on time perception, Blount and Janicik (2001) distinguished between delay and hastening as positive (t_0_ + Δt) and negative deviation (t_0_ − Δt) from an expected time point (t_0_), respectively. Similarly, Chu and Choi (2005) describe *non-procrastination (a low level of procrastination)* as finishing a task by evenly distributing allocated time (t_n_), where *precrastination* reflects accelerated task completion early in the timeline (t_n_ − Δt). Reverse-coded procrastination items capture non-procrastination (e.g., “I do my work when I plan to do it”) but not the urgency underlying precrastination (Chu and Choi, 2005). As such, conflating the two constructs obscures the unique motivational and behavioral features of precrastination and limits our understanding of its potential organizational impact.

Third, the current literature fails to capture the multifaceted nature of precrastination. We propose that precrastination can occur at multiple stages in the task completion process: immediate start, rapid progress, or early completion. Although prior studies have acknowledged this complexity, they have done so in a fragmented manner. Rosenbaum et al. (2014, p. 1487) defined precrastination as “the tendency to *complete*, or at least *begin*, tasks as soon as possible,” while later work described it as “the desire to *progress* on tasks as soon as possible,” (Rosenbaum and Sauerberger, 2019, p. 4). These variations highlight the need for greater conceptual clarity (Fournier et al., 2019) and better psychometric evidence of the construct in the workplace setting (Gehrig et al., 2023). Thus, it is critical to incorporate its distinct yet interrelated dimensions of precrastination to advance theory and measurement in the organizational context.

Fourth, despite the growing interest in precrastination, a psychometrically sound measure suited for workplace contexts is lacking. A recent effort by Gehrig et al. (2023) represents an important initial step; however, several limitations suggest opportunities for refinement. For example, the study does not clearly define the construct—a foundational step in scale development (Podsakoff et al., 2016)—nor does it detail item development or validity testing procedures. Moreover, some items appear to measure *emotional responses* (e.g., “Starting a task early gives me a relieving feeling”) or *ideological beliefs* about precrastination rather than the behavioral tendency to precrastinate (e.g., “Starting a task early is more important to me than finishing it early”), which begs questions about potential confounding variables and the underlying factor structure of the precrastination measure.^1^ Additionally, this study was conducted using a sample of adolescents, a population that may not effectively provide inferences about organizational phenomena (DeShon, 2002). Given the existing limitations in defining and operationalizing precrastination in Gehrig et al. (2023), we propose a more comprehensive, theory-driven conceptualization tailored to organizational contexts. In the sections that follow, we introduce a definition of workplace precrastination, provide its theoretical foundations, and clarify its organizational relevance.

### 1.2 Definition of workplace precrastination

We define *workplace precrastination* (hereafter referred to as *precrastination*) as *the tendency of an employee to initiate, make rapid progress, and complete official tasks well in advance of the organizational expectations or deadlines*. Precrastination is characterized by three key aspects: (1) precrastination is an *individual difference* var*iable* influenced by both trait and situational factors; (2) it manifests when tasks involve *deadlines*; and (3) the target task in precrastination is *officially assigned by the organization*. These aspects are elaborated upon in the discussion below.

We define precrastination as an individual difference variable that can be influenced by various individual and task-related predictors. A recent review of precrastination research suggests that both individual and environmental factors can affect precrastination (Rosenbaum et al., 2019). Drawing on the trait activation theory (Tett et al., 2021), we position precrastination as an individual difference rooted in certain personality traits, but responsive to task demands. This view aligns with prior research showing that precrastination is linked to stable personality traits, such as conscientiousness; for instance, Sauerberger (2019) found that 22% of participants consistently chose the precrastinating option across repeated trials. Although contextual factors such as task difficulty and weight can shape precrastinatory behavior (Fournier et al., 2019), these situational effects likely moderate, rather than define, the underlying predisposition to precrastinate.

To define what constitutes “sooner than expected,” it is crucial to articulate the meaning of “expectation.” In organizational settings, employees manage their activities around salient temporal signals, such as project deadlines, which allocate a finite timeframe for task completion (Blount and Janicik, 2001). Precrastination involves a deviation from this expected pacing, occurring when individuals rush to initiate or complete tasks ahead of such referent points. We therefore propose that an explicit deadline is a crucial anchor for determining whether a particular course of action qualifies as precrastination.

In our conceptualization, precrastination represents a tendency to complete formally assigned tasks, excluding non-work-related tasks. We consider the organizational context as a crucial boundary condition for this construct, distinguishing our approach from prior studies conducted in experimental, non-organizational contexts (Blinch and DeWinne, 2019; Fournier et al., 2019). By focusing on officially assigned tasks, our definition aligns with the idea that precrastination expresses as an individual difference to a contextual need.

### 1.3 Three dimensions of workplace precrastination

Organizational theories of time suggest that human tendencies unfold across three distinct temporal stages (Ancona et al., 2001; George and Jones, 2000; Mohammed and Nadkarni, 2011). These three stages are: a starting point, a middle part, and an endpoint. Building on this perspective, we propose that workplace precrastination is reflected in three interrelated, yet distinct, dimensions that align with the three temporal stages mentioned above: (1) immediate start, (2) rapid progress, and (3) early completion. Below we describe how the three dimensions correspond to the three broad temporal stages. We also explain why the dimensions may be correlated yet reflect theoretically distinct aspects of precrastination.

#### 1.3.1 Immediate start

The first component of our definition entails an *immediate start*, which reflects a tendency of precrastinators to start working on the assigned task as soon as it is given to them.

This dimension of precrastination corresponds with the starting point of the temporal process (Mohammed and Nadkarni, 2011). Prior research describes this tendency as “a strong inclination to start the task (or sub-goal) as soon as possible” (Fournier et al., 2019, p. 1674) or engage in “starting a sub-goal sooner or bringing one’s current state closer to the end goal state” (Villarreal, 2020, p. 3). We conceptualize this phase as a *self-starting, reactive tendency* characterized by the rapid beginning of task-related activities. Although the tendency to take the initiative also appears in constructs like personal initiatives and proactive work behavior (Frese et al., 1997), precrastination is separated by its *reactive* nature—often triggered by the perception of time pressure or the urgency of approaching deadlines (Zhu et al., 2018).

#### 1.3.2 Rapid Progress

The second component of the precrastination tendency is *rapid progress*, which reflects the tendency of precrastinators to attempt speedy execution of the assigned task. Rapid progress is exhibited *during* the task-completion process. This dimension of precrastination corresponds with the middle stage of the temporal process because it happens after the process has been initiated but has not been completed (Mohammed and Nadkarni, 2011). Rapid progress may have its roots in the notion of commitment escalation: individuals persist in exerting continued (and often excessive) effort toward their endeavors, even when those endeavors are sub-optimal, either due to impulsive initiation or a desire for quick completion (Moon, 2001). Precrastinators may feel compelled to sustain their pace due to prior investment (“sunk efforts”) or fear of abandoning the task midway, even if this rapid option is more taxing. Related concepts in other fields echo this phase: economists describe “impatience” as prioritizing immediate tasks while discounting the utility of more distant ones (O’Donoghue and Rabin, 1999), and the “mere urgency effect” captures the compulsion to act quickly based on perceived time pressure, even when better options exist (Zhu et al., 2018).

#### 1.3.3 Early completion

The third component of precrastination, *early completion,* represents precrastinators’ tendency to complete tasks sooner than necessary (Rosenbaum and Sauerberger, 2019; VonderHaar et al., 2019). The early completion dimension reflects the tendency to reach the end goal as quickly as possible. Hence, this dimension represents how quickly a task reaches the *endpoint* of the temporal process (Mohammed and Nadkarni, 2011). Early completion can be understood in terms of the need for closure—a preference for quick decisions, rapid arrival at a solution, and an aversion toward uncertainty (Kruglanski, 1989). Accelerating task completion can be psychologically rewarding, offering a sense of control and satisfaction by enabling individuals to “check off” tasks from their mental agendas (Rosenbaum et al., 2014). Empirical evidence supports this pattern: VonderHaar et al. (2019) demonstrated that participants chose to complete memory retrieval tasks more quickly, even at the expense of accuracy, reinforcing the motivational pull toward early task closure.

Taken together, the three dimensions map onto different time points of task performance: initiation (starting point), process (middle stage), and completion (endpoint) (Ancona et al., 2001; George and Jones, 2000; Mohammed and Nadkarni, 2011). We believe that distinguishing among these three dimensions of workplace precrastination clarifies how precrastination unfolds across time: they appear at different stages of task progress, yet all represent manifestation of the same underlying tendency to precrastinate.

Further, we argue that although immediate start, rapid progress, and early completion are likely to be correlated, each captures a unique temporal manifestation of workplace precrastination. For example, some individuals may begin working immediately, but slow down later, while others may take some time to begin working, but once they start, they may complete the task quickly. Thus, workplace precrastination is best understood as a reflective construct composed of these three manifestations of the underlying tendency to precrastinate. This conceptualization allows for a more nuanced understanding of how each dimension of precrastination can emerge at different points in the task cycle.

### 1.4 Hypothesis development

In this section, we developed and tested two sets of hypotheses to examine the validity of the precrastination scale. The first set of hypotheses pertained to testing its discriminant validity with procrastination (H1), workaholism (H2), positive and negative affect (H3a, H3b), and regulatory focus (H4a, H4b). The second set of hypotheses proposed potential antecedents and outcomes of workplace precrastination. We included antecedent variables such as conscientiousness (H5), personal initiative (H6), trait anxiety (H7), and task characteristics (H8a, H8b), and focused on two categories of outcome variables: task and citizenship performance (H9a, H9b) and attitudes (H10a, H10b, H10c). Further, we conducted incremental validity testing of our workplace precrastination scale over the established procrastination scales on these outcome variables.

To establish discriminant validity, we first tested whether precrastination and procrastination are distinct constructs. Procrastination is widely characterized as a form of self-regulatory failure involving the delay of an intended action (Steel, 2007, 2010). This failure to carry out planned actions is inherently a deficiency. Consequently, a low level of procrastination is generally viewed positively, as it implies self-control in bridging the intention-action gap (Chu and Choi, 2005). Precrastination, however, is not entirely advantageous and reflects a distinctive feature marked by prompt and automatic initiation of, proceeding on, or completion of tasks. This is quite distinct from the delaying of pre-established plans in procrastination (Steel, 2007).

Further, impulsivity, that is, starting to work quickly but without adequate thought, could be an indication of precrastination (Zermatten et al., 2005). This form of impulsivity indicates the urge to complete the task early to release working memory and feel at ease (Patterson and Kahan, 2020). However, the form of impulsivity most often tied to procrastination is avoidance-impulsivity: when a task is perceived as aversive, avoidant-impulsive individuals impulsively divert attention to more immediately gratifying activities to regulate mood, thereby delaying the task completion (Steel, 2007). Taken together, precrastination and procrastination map onto different impulsive propensities with distinct mechanisms and implications, which further suggests that they are distinctive constructs.

Accordingly, we expect a significant negative correlation between the two constructs, but not one so high as to imply redundancy (e.g., correlations of 0.80 or above; Kline, 2005).

*H1*: Precrastination negatively correlates, but does not show redundancy, with procrastination.

Workaholism is a related but conceptually distinct construct from precrastination. Characterized as an addiction to work, workaholism denotes the behavior individuals who are highly involved in work, feel compelled to work, and tend not to enjoy life outside of work (Clark et al., 2020). Although precrastination may also involve a compulsive element, it is more externally triggered, typically by the presence of an officially assigned task. Precrastination also diverges from workaholism in the form of excessive work engagement. In precrastination, excess means working speedily to offload tasks despite the extra effort (Rosenbaum et al., 2014), while workaholism involves working beyond what is reasonably required. As such, we expect that precrastination positively relates to workaholism but remains distinct from it.

*H2*: Precrastination positively correlates, but does not show redundancy, with workaholism.

We include trait positive and negative affect to examine the emotional landscape associated with precrastination. As precrastination involves early initiation or rapid sub-goal completion, it can elicit a sense of accomplishment and energy. Research suggests precrastination is linked to positive emotions stemming from the anticipation of completing tasks ahead of schedule (Patterson and Kahan, 2020). Behavioral psychologists describe this as the pursuit of end-state comfort (Wasserman, 2019), or relief from task-related cognitive load (VonderHaar et al., 2019). Conversely, precrastination might be negatively associated with negative affect. Research suggests that precrastination reduces the cognitive load of remembering pending tasks (Rosenbaum et al., 2019), thus freeing up mental resources and reducing task-induced stress. In the absence of precrastination behavior, negative feelings might arise due to the anxiety and mental strain triggered by approaching deadlines.

*H3*: Precrastination (a) positively correlates with positive affect, and (b) negatively correlates with negative affect.

Self-regulation theory posits the coexistence of two systems: (1) promotion focus, which involves striving toward desired goals, and (2) prevention focus, which centers on averting undesired consequences (Higgins, 1997). Individuals with a promotion focus typically regulate their actual selves to align with their ideal selves by addressing growth, development, and exploration needs. Like promotion-focused individuals, precrastinators exhibit a forward-leaning, outcome-driven orientation, marked by a desire to act quickly and efficiently to achieve success. Prevention focus manifests as the reduction of discrepancies between the actual self and the ideal self by fulfilling the needs of security, safety, and obligations (Higgins, 1997). Precrastination mirrors the activities associated with a prevention focus in which there is an inner compulsion to correct errors or avoid potential losses to complete the task before the deadline.

*H4*: Precrastination positively correlates with (a) promotion regulatory focus and (b) prevention regulatory focus.

Previous studies on precrastination have shown a relatively stable tendency for a certain percentage of students to make precrastinatory choices across different experimental trials (59% in Sauerberger et al., 2018; 22% in Sauerberger, 2019). This consistent behavioral manifestation of precrastination may be attributed to dispositional antecedents such as conscientiousness. Conscientiousness is a broad personality term that includes different facets such as self-discipline, deliberation, dutifulness, competence, and striving for achievement (Costa and McCrae, 1992). We posit that conscientiousness may be an important antecedent factor in initiating precrastination. Specifically, individuals with a high level of conscientiousness exhibit self-discipline, which enables them to begin a task and carry it through to completion despite other distractions. This tendency toward self-discipline possibly facilitates precrastination. Further, conscientious individuals are generally self-driven (Judge et al., 1997), so their innate sense of duty and discipline would motivate them to start working on the task quickly and to maintain a commitment to its early completion.

*H5*: Conscientiousness is positively related to precrastination.

Personal initiative, defined as a proactive and self-starting approach to work, is another possible antecedent of precrastination. Individuals with high levels of personal initiative tend to take charge of situations, act to exert control, and move quickly to address tasks before they become urgent (Frese et al., 1997). This proactive behavior aligns closely with the concept of precrastination, because both constructs indicate an action-oriented approach to organizational tasks (Hong et al., 2016). Moreover, personal initiative fosters a sense of control and efficacy, which may motivate individuals to complete tasks ahead of time. Individuals with high personal initiative have high self-efficacy, intrinsic motivation, and positive affect (Hong et al., 2016), which in turn possibly boosts confidence and encourages greater precrastination.

*H6*: Personal initiative is positively related to precrastination.

General trait anxiety represents a dispositional tendency to experience feelings such as nervousness, uneasiness, and tension (Cheng and McCarthy, 2018). Research has suggested that a high degree of trait anxiety can promote risk-aversion, punctuality (Izard and Youngstrom, 1996), and an increased sensitivity to surroundings (Elliot and McGregor, 1999). Given the hyper-vigilance associated with trait anxiety, we propose that it may predispose individuals to perceive situations as more threatening or demanding, thus leading to greater discomfort with task incompletion and, consequently, to precrastination. A growing body of organizational research has highlighted the facilitative role of trait anxiety and suggested that anxiety is an alert mechanism to signal discrepancies between desired and actual progress toward goal completion, which can drive increased effort and engagement in tasks (Cheng and McCarthy, 2018). In line with this, we argue that the anticipatory worries associated with tasks may motivate employees to act quickly upon their tasks to alleviate their anxiety.

*H7*: Trait anxiety is positively related to precrastination.

We propose that precrastination may arise due to contextual demands at work. Specifically, we examine two task characteristics likely to influence precrastination: task interdependence and task autonomy (Morgeson and Humphrey, 2006). Task interdependence indicates the extent to which one’s job relies on others and is suggested to create highly engaged work environments that necessitates rapid information exchange and greater coordination. In such settings, the need for rapid responses may increase as the completion of each member’s task depends on the accomplishments of other members. Because interdependence may provide a clear structure for task allocation and member interactions, it helps manage task uncertainty and ambiguity and enables members to act promptly on tasks.

Task autonomy, the freedom and discretion in conducting a task (Hackman and Oldham, 1975), may also promote precrastination. Task autonomy often enhances ownership and responsibility, potentially motivating individuals complete tasks early to maintain a sense of control and accomplishment. The ability to prioritize and manage tasks independently can create a psychological drive to complete tasks ahead of time, especially when early completion is interpreted as a sign of efficiency or diligence.

*H8*: (a) Task interdependence and (b) task autonomy are positively related to precrastination.

Task performance, or in-role performance, is a critical outcome variable in organizational behavior literature. It includes meeting or surpassing quantitative and qualitative performance standards, fulfilling duties and responsibilities, and displaying technical expertise (Borman and Motowidlo, 1997). Given that precrastination is characterized by task-oriented behavioral tendencies with a strong emphasis on early task completion, we expect that precrastination would be positively related to task performance.

Citizenship behavior is another type of performance that refers to individuals performing helpful tasks that are not a part of their official responsibilities (Borman and Motowidlo, 1997). We posit that precrastination would be positively related to citizenship performance, because precrastinators are proactive in initiating, executing, and completing tasks quickly, which can indirectly help coworkers and the larger organization. In addition, precrastinators may go above and beyond what is required because they may have extra time to help other colleagues as a result of finishing their own work ahead of time.

We next examine whether precrastination predicts performance outcomes beyond what is accounted for by procrastination. While procrastination is consistently associated with poor performance (Steel, 2007), our goal is to establish the incremental validity of precrastination by testing its unique contribution on performance. Beyond the avoidance of unnecessary delays, we propose that precrastination may contribute to organizational performance by enabling early task completion, promoting resource flexibility, and creating space for innovation.

*H9*: Precrastination is positively related to and adds unique variance to the prediction of (a) task performance and (b) organizational citizenship behavior after controlling for the effect of procrastination.

Attitudes evolve as a consequence of the emotional and cognitive responses linked to an object. Precrastinators are likely to experience a sense of positive affect when immediately engaging in tasks, so it is reasonable to anticipate that precrastination has a positive impact on job-related attitudes. It is also often characterized as cognitively rewarding, as completing tasks promptly can provide mental relief (Patterson and Kahan, 2020; Rosenbaum et al., 2014). Given the positive affect and cognitive benefits associated with precrastination, we hypothesize that precrastination is positively associated with favorable workplace attitudes.

Within the category of attitudes, we examined three constructs frequently studied in the workplace. First, *job satisfaction*, which is characterized by the extent to which employees experience positivity and pleasure in their job roles (Hackman and Oldham, 1975), is of particular interest. It is plausible that precrastinators may exhibit a heightened level of job satisfaction, as they may complete their official tasks quickly, which provides them with a sense of satisfaction and achievement (Ng et al., 2007).

Second, *work engagement* pertains to the state of a high level of personal involvement and the presence of positive affect in the workplace (Bledow et al., 2011). Although precrastinators may not necessarily experience intrinsic interest or enjoyment in the work itself, their active involvement in their tasks may be a precursor to work engagement. More precisely, precrastinators are excessively focused on task completion, and this involvement may extend to their engagement in the workplace (Blinch and DeWinne, 2019). Given the underlying high degree of involvement with the task at hand—and, by extension, the entire set of work responsibilities—we expect precrastination to be positively associated with work engagement.

Third, *organizational commitment* is the bond that an employee feels toward the organization (Meyer and Herscovitch, 2001). Precrastinators’ desire to complete their official tasks quickly can be seen as their commitment to task accomplishment. By definition, these tasks are assigned by their organizations. Thus, precrastination can lead to the development of a bond with the organization. We therefore propose that precrastination is associated with organizational commitment.

Additionally, we propose that precrastination may improve our ability to predict these attitudinal outcomes over and above procrastination. To date, research has suggested that procrastination is negatively related to organizational attitudinal outcomes (Steel, 2007). Given the unique emphasis of precrastination on early task initiation, progress, and completion, we anticipate that precrastination would add unique predictive variance beyond that captured by procrastination for these attitudinal variables.

*H10*: Precrastination is positively related to and adds unique variance to the prediction of (a) job satisfaction, (b) work engagement, and (c) organizational commitment, after controlling for the effect of procrastination.

The purpose of this research is to develop and validate a set of scales to advance our understanding of workplace precrastination. With this purpose in mind, we conducted our research through two studies. In Study 1, we used three samples to develop items for the three-dimensional precrastination scale using the above definition. In Study 2, using a multi-wave sample, we tested scale validity and proposed the possible antecedents and outcomes of workplace precrastination.

## 2 Workplace precrastination scale development (study 1)

### 2.1 Item generation (step 1)

We generated an initial pool of precrastination items following a deductive item-generation approach (Hinkin, 1998) to reflect our definition of precrastination and its dimensions. To begin with, we thoroughly examined the existing measures on precrastination and related constructs (e.g., procrastination, Type A). Scales reviewed in this step were included in the Appendix B. The first author drew from a review of these scales and wrote an initial item pool of precrastination consisting of 47 items in total.

Next, the second and the third authors independently sorted all items into categories. Items were eliminated if they were too general, contained language that confounded emotions and consequences, or were unclear. The agreement between the two coders in sorting categories and eliminating items was high (*r* > 0.90). The few remaining discrepancies were thoroughly discussed, which led to a final consensus that resulted in three categories aligned with the three dimensions proposed. For each of the three dimensions, seven items were created by using the most clearly written and frequently mentioned items in that category. This process led to our initial item pool, which consisted of 21 items.

### 2.2 Item review (step 2)

During the item review step, we asked 43 undergraduate students (Sample 1) from a mid-sized Southeast U. S. university to review the 21 items we had generated in the previous step. Most participants were male (83.3%), Caucasian (66.7%), with an average age of 25 years (SD = 5.0). About 75% of the participants were currently employed, with 41.7% in full-time positions and 36.1% in part-time roles. On average, participants reported working 31.5 h per week (SD = 13.2) and had an average of 7.2 years of work experience (SD = 5.2).

Consistent with Bennett and Robinson’s (2000) approach, participants were asked to rate the extent to which each item met three criteria on a 5-point scale. The first criterion was *consistency*, which measured the degree to which each item was consistent with the provided definition of precrastination (1 = *very inconsistent*; 5 = *very consistent*). The second criterion was *clarity*, which measured the degree to which the wording of each item was clear and understandable (1 = *very unclear*; 5 = *very clear*). The last criterion was frequency, which measured the frequency with which each item takes place in the workplace (1 = *rarely*; 5 = *almost always*). Participants were also asked to provide comments and feedback on the initial item pool in an open-ended format.

Although all items received a mean score of 3.0 or higher on the rating criteria (Consistency *M* = 3.9, SD = 0.2, Clarity *M* = 3.6, SD = 0.4; Frequency *M* = 3.8; SD = 0.2), we conducted a comparative analysis of the mean scores for each item to further eliminate items that obtained relatively low scores within each subdimension of precrastination. For example, the item “I start working from the first minute to complete an assigned task” in the immediate start subdimension was scored at 3.1, and the item “As soon as I am given a task, I work very speedily to complete my tasks” in the rapid progress subdimension was scored 3.0. In total, 3 items were eliminated in this step, resulting in a pool of 18 items.

We further refined the initial item pool based on the open-ended feedback provided. Participants identified seemingly redundant phrases that made the scale wordy and repetitive. For example, all the initial items contained redundant and repetitive phrases such as “Once a task is given to me…,” “As soon as I am given a task…,” “As soon as a task is assigned to me…,” or “When I am given a task….” Accordingly, we decided to remove the seemingly redundant phrases and selected one general phrase (i.e., “As soon as I am given a task…”), which made the items shorter and less cognitively demanding (Hinkin, 1998).

### 2.3 Item reduction (step 3)

In the item reduction step, we used the slightly modified items from the item review step above to reduce the scale length. Participants in this step were undergraduate students from a mid-sized southeast U. S. university (Sample 2). A total of 204 individuals participated in the survey, out of which 200 complete responses were included in the analysis. Most were male (65.5%) and white (61.5%), with an average age of approximately 23.6 years (SD = 6.0). About 73.5% of the respondents reported current employment, with an average of 29.5 h worked per week (SD = 12.3) and an average of 2.4 years of experience in their current positions (SD = 3.4).

We employed exploratory factor analysis (EFA, maximum likelihood with Promax rotation) to investigate the underlying factor structure of items using Sample 2. Our analysis identified three distinct factors (minimum eigenvalue = 1.52; total variance explained = 79.7%). As expected, all items exhibited strong loadings on the respective factors (with factor loadings ranging from 0.75 to 0.98). From these results, we retained all 18 items to constitute our precrastination scale: *Immediate Start* (hereafter, IS: 6 items), *Rapid Progress* (hereafter, RP: 6 items), and *Early Completion* (hereafter, EC: 6 items). We believe this three-factor model effectively encapsulates the primary behavioral facets of precrastination and offers a concise and conceptually sound framework for understanding and measuring the construct. The items and the factor loadings for the three-factor solution are reported in Table 1.

**Table 1:** Study 1 (Step 3): exploratory factor loadings of workplace precrastination items.

Item	Dimensions		
*As soon as I am given a task …*	Immediate start	Rapid progress	Early completion
Immediate start (IS)			
1. I quickly jump on it to finish it.	**0.747**	0.098	0.112
2. I rush to start working on it.	**0.863**	0.046	−0.045
3. I begin working on it immediately.	**0.943**	0.013	−0.003
4. I start working on it right away.	**0.975**	−0.082	0.021
5. I act upon it immediately.	**0.875**	0.019	0.043
6. I quickly start working on it.	**0.910**	0.030	0.030
Rapid progress (RP)			
7. I work at a rapid pace.	0.038	**0.898**	−0.024
8. I expedite my working speed.	0.055	**0.902**	0.065
9. I work rapidly to complete it.	−0.065	**0.913**	−0.102
10. I move quickly to complete it.	−0.232	**0.766**	−0.046
11. I make fast progress on it.	0.048	**0.812**	0.157
12. I work fast to complete it.	0.003	**0.892**	−0.005
Early completion (EC)			
13. I complete my task with a lot of time to spare.	−0.075	0.065	**0.793**
14. I finish my task sooner than necessary.	−0.036	−0.067	**0.884**
15. I finish my task much before the deadline.	−0.111	0.036	**0.759**
16. I complete my task earlier than required.	0.035	0.049	**0.910**
17. I complete my task much ahead of the deadline.	−0.087	−0.038	**0.843**
18. I complete my task earlier than needed.	0.099	0.007	**0.930**

*N* = 200. Factor loadings above 0.70 are in bold.

### 2.4 Confirmatory factor analysis (step 4)

In this step, we sought to confirm the factor structure identified in the previous step using Confirmatory Factor Analysis (CFA). A sample of working professionals (Sample 3) was recruited through Prolific for this purpose. Participants were asked to respond to Wave 1 and Wave 2 surveys 2 weeks apart. We used 291 responses on the precrastination measure from Wave 1 to test the psychometric properties of our scale. Nearly two thirds (63.2%) of the participants were male, and 74.2% were Caucasian (mean age = 38.3, SD = 10.2 years; mean organization tenure = 6.6 years, SD = 5.6 years). Most participants had received a bachelor’s degree or higher in college (82.13%), with 62.54% holding a supervisory position. Participants were employed in a variety of industries, including banking (5%), construction (4%), customer service (4%), education (13%), health care (14%), information technology (13%), retail (6%), sales (8%), and social services (2%).

We performed CFA using R software with the lavaan (latent variable analysis; Rosseel, 2012) package. We sought to confirm the three-factor structure suggested by the EFA above. The three-factor model was a well-fitting model (CFI = 0.94; TLI = 0.93; SRMR = 0.046; RMSEA = 0.097). Factor loadings ranged from 0.745 to 0.944 (*M* = 0.881), and all indicators loaded onto the intended factor (*p* < 0.001). We also ran alternative two-factor (CFI = 0.74; TLI = 0.70; SRMR = 0.137; RMSEA = 0.204) and one-factor (CFI = 0.61; TLI = 0.56; SRMR = 0.132; RMSEA = 0.246) models. These models had weaker fit than the hypothesized 3-factor model, supporting the underlying three-dimensional structure of precrastination.

With the evidence of acceptable factor structure, we then tested the psychometric properties of the scale. The alpha coefficient reliability estimates were constantly high for responses across two waves (*M* = 0.96). Composite reliabilities for the three dimensions were high (0.95, 0.96, and 0.95). Intercorrelations among the three dimensions were all positive and significant (IS and RP: $\bar{r}$ = 0.68, *p* < 0.001; IS and EC: $\bar{r}$ = 0.55, *p* < 0.001; RP and EC: $\bar{r}$ = 0.60, *p* < 0.001). Taken together, these results indicated that the three dimensions of precrastination are highly correlated with one another but assess different aspects of the construct. In the next phase, we developed hypotheses to test the validity of the workplace precrastination scale.

## 3 Validate workplace precrastination scale (study 2)

### 3.1 Participants

We used Sample 3 (working adults recruited from Prolific) to test the validities and preceding hypotheses. Data from Wave 1 and Wave 2 were employed in this study. The Wave 1 survey contained items for precrastination, procrastination, workaholism, positive and negative affect, regulatory style, conscientiousness, personal initiative, trait anxiety, and task characteristics. The Wave 2 survey measured precrastination, task performance, citizenship performance, job satisfaction, work engagement, and organizational commitment. With prescreening criteria (e.g., aged 18–65 and full-time employment status, from the U. S.), 291 participants completed the survey at Wave 1. A total of 286 participants completed the Wave 2 survey (98.28% of retention rate).

### 3.2 Measures

Unless otherwise indicated, all measures were rated on a 5-point Likert scale ranging from strongly disagree to strongly agree.

#### 3.2.1 Precrastination (waves 1 and 2)

Newly developed precrastination items from Study 1 listed in Table 1 were used.

#### 3.2.2 Procrastination (wave 1)

Procrastination was measured using the 9-item Irrational Procrastination Scale (IPS; Steel, 2010) and 12-item Pure Procrastination Scale (PPS; Svartdal and Steel, 2017). Sample items from the IPS include “I delay tasks beyond what is reasonable” and “I do not do anything when it needs to be done.” Example items from the PPS are “I am not very good at meeting deadlines” and “I delay making decision until it’s too late.”

#### 3.2.3 Workaholism (wave 1)

We used the 16-item Multidimensional Workaholism Scale (MWS; Clark et al., 2020). Sample items include “I have a strong inner desire to work all the time (motivational); “In general, I spend my free time thinking about work” (cognitive); “I feel upset if I cannot continue to work” (emotional); and “I work more than what is expected of me” (behavioral).

#### 3.2.4 Positive affect and negative affect (wave 1)

Trait positive and negative affect were assessed using the 20-item Positive and Negative Affect Schedule (PANAS; Watson et al., 1988). Participants rated their general tendency to experience positive affect (e.g., determined, inspired, active) or negative affect (e.g., upset, nervous, guilty).

#### 3.2.5 Regulatory focus (wave 1)

Regulatory focus was measured with the 12-item Regulatory Focus at Work scale (Wallace and Chen, 2006). Sample items for *promotion focus* include “accomplishing a lot at work” and examples of *prevention focus* include “following the rules and regulations”.

#### 3.2.6 Conscientiousness (wave 1)

Conscientiousness was measured with the 12-item NEO Five-Factor Inventory (Costa and McCrae, 1992). Sample items include “I keep my belongings clean and neat” and “I work hard to accomplish my goals”.

#### 3.2.7 Personal initiative (wave 1)

Personal initiative was measured using a 7-item scale developed by Frese et al. (1997). Example items include “I actively attack problems” and “I use opportunities quickly in order to attain my goals”.

#### 3.2.8 Trait anxiety (wave 1)

Trait anxiety was assessed by the 7-item scale from Spitzer et al. (2006). Participants were asked how often they have been bothered by specific problems over the last 2 weeks (0 = *not at all* to 3 = *nearly every day*). A sample item from this scale is “feeling nervous, anxious or on edge”.

#### 3.2.9 Task characteristics (wave 1)

The 15-item Work Design Questionnaire (WDQ) was employed to measure task characteristics (Morgeson and Humphrey, 2006). An example item for task interdependence is “other jobs depend directly on my job,” and an item from task autonomy dimension is “my job allows me to plan how I do my work”.

#### 3.2.10 Task performance (wave 2)

Task performance was measured with five items from the In-Role Behavior scale (IRB; Williams and Anderson, 1991). Sample items include “I adequately complete assigned duties” and “I perform tasks that are expected of me”.

#### 3.2.11 Citizenship behavior (wave 2)

Citizenship behavior was assessed with seven items using the organizational citizenship behavior scale by Dalal et al. (2009). Example items are “I go out of my way to be a good employee” and “I display loyalty to the organization”.

#### 3.2.12 Job satisfaction (wave 2)

Job satisfaction was measured with three items using the Job Diagnostic Survey (JDS; Hackman and Oldham, 1975). Sample items include “Generally speaking, I am very satisfied with my job” and “In general, I like my job”.

#### 3.2.13 Work engagement (wave 2)

The 8-item Utrecht Work Engagement Scale (UWES-9; Schaufeli et al., 2006) was used to measure work engagement. Participants rated how frequently they felt the way indicated in each item about their work in the past month on a 5-point scale (1 = *never*, 5 = *always*). Sample items include “I am immersed in my work” and “I am enthusiastic about my job”.

#### 3.2.14 Organizational commitment (wave 2)

A unidimensional commitment scale with four items was used to assess organizational commitment (Klein et al., 2014). Sample items include “I am committed to my organization” and “I care about my organization”.

#### 3.2.15 Control variable (wave 1)

We controlled for procrastination due to its correlation with organizational outcomes (Legood et al., 2018; Yang et al., 2023), and 21 items developed by Steel and colleagues were included as controls (Steel, 2010; Svartdal and Steel, 2017).

### 3.3 Discriminant validity results (step 1)

Bivariate correlations and reliabilities among all study variables are reported in Table 2. We hypothesized (H1) that precrastination and its dimensions would exhibit negative correlations with procrastination but without redundancy. As anticipated, precrastination displayed negative correlations with both procrastination measures: IPS ( $\bar{r}$ = − 0.58, *p* < 0.001) and PPS ( $\bar{r}$ = − 0.53, *p* < 0.001). Moreover, all dimensions of precrastination showed negative significant relationships with procrastination (IS: $\bar{r}$ = − 0.53, *p* < 0.001; RP: $\bar{r}$ = − 0.37, *p* < 0.001; EC: $\bar{r}$ = − 0.56, *p* < 0.001).

**Table 2:** Study 2: descriptive statistics, correlations, and reliabilities for validity testing constructs.

Variable	*M*	SD	1	2	3	4	5	6	7	8	9	10	11	12	13	14	16	17	18	19	20	21	22	23	24
1 PRE	3.89	1.01	**0.96**																						
2 PRE – IS	3.74	1.05	—	**0.95**																					
3 PRE – RP	3.54	1.02	—	0.70	**0.96**																				
4 PRE – EC	3.66	1.01	—	0.53	0.60	**0.95**																			
5 PRO	2.14	1.22	−0.53	−0.53	−0.37	−0.53	**0.97**																		
6 WORH	2.82	1.39	0.25	0.19	0.23	0.16	0.08	**0.94**																	
7 PANAS – P	3.90	1.02	0.46	0.46	0.33	0.37	−0.48	0.25	**0.92**																
8 PANAS – N	1.82	1.08	−0.34	−0.27	−0.23	−0.34	0.52	0.07	−0.58	**0.93**															
9 REG – M	3.59	0.95	0.55	0.51	0.46	0.37	−0.29	0.34	0.45	−0.24	**0.88**														
10 REG – V	4.29	0.81	0.28	0.30	0.20	0.21	−0.36	0.12	0.35	−0.23	0.53	**0.89**													
11 CONS	4.17	0.92	0.59	0.52	0.42	0.55	−0.80	0.19	0.69	−0.53	0.46	0.48	**0.93**												
12 PER – I	3.93	0.97	0.62	0.51	0.50	0.51	−0.55	0.33	0.73	−0.48	0.52	0.36	0.79	**0.88**											
13 TACI	3.36	1.20	0.17	0.11	0.18	0.15	−0.04	0.03	0.01	−0.11	0.11	0.03	0.03	0.08	**0.89**										
14 TACA	4.00	0.95	0.16	0.17	0.12	0.12	−0.15	0.09	0.19	−0.21	0.22	0.24	0.24	0.25	0.05	**0.95**									
16 ANX	2.19	1.15	−0.23	−0.19	−0.14	−0.26	0.52	0.17	−0.52	0.80	−0.16	−0.18	−0.44	−0.39	−0.14	−0.25	**0.94**								
17 GEN	0.61	0.49	−0.02	−0.05	0.04	−0.06	0.08	−0.03	0.02	−0.06	−0.07	−0.09	−0.10	0.01	0.11	0.09	−0.15								
18 AGE	38.29	10.2	0.04	0.12	0.00	0.04	−0.22	0.05	0.06	−0.11	0.07	0.20	0.20	0.13	0.05	0.14	−0.14	−0.17							
19 TEN	6.63	5.57	0.07	0.13	0.03	0.03	−0.18	0.07	0.08	−0.09	0.13	0.24	0.20	0.11	0.09	0.18	−0.11	−0.13	0.44						
20 PERF	4.70	0.56	0.18	0.15	0.11	0.19	−0.40	−0.08	0.27	−0.33	0.19	0.40	0.49	0.33	0.01	0.22	−0.28	−0.05	0.15	0.08	**0.85**				
21 OCB	4.31	0.84	0.34	0.33	0.21	0.27	−0.32	0.14	0.47	−0.28	0.54	0.46	0.56	0.57	0.04	0.28	−0.20	−0.17	0.19	0.17	0.49	**0.82**			
22 SAF	4.00	1.09	0.29	0.31	0.20	0.17	−0.32	0.21	0.52	−0.37	0.46	0.34	0.48	0.38	0.02	0.44	−0.33	−0.01	0.08	0.08	0.23	0.53	**0.95**		
23 ENG	3.43	1.08	0.39	0.40	0.29	0.26	−0.35	0.35	0.68	−0.37	0.62	0.41	0.54	0.55	−0.02	0.36	−0.33	0.01	0.12	0.10	0.19	0.57	0.82	**0.94**	
24 COMM	3.80	1.07	0.28	0.31	0.22	0.12	−0.20	0.35	0.51	−0.25	0.50	0.35	0.39	0.41	0.02	0.38	−0.20	−0.07	0.14	0.14	0.12	0.56	0.82	0.80	**0.96**

Sample 3 (Wave 1) = 291. Sample 3 (Wave 2) = 286. PRE, precrastination; PRE – IS, precrastination immediate start; PRE – RP, precrastination rapid progress; PRE – EC, precrastination early completion; PRO, procrastination; WORH, workaholism; PANAS – P, positive affect scale; PANAS – N, negative affect scale; REG – M, promotion regulatory style; REG – V, prevention regulatory style; CONS, conscientiousness; PER – I, personal initiative; TACI, task interdependence; TACA, task autonomy; ANX, trait anxiety; GEN, gender; TEN, tenure; PERF, task performance; OCB, organizational citizenship behavior; SAF, job satisfaction; ENG, work engagement; COMM, organizational commitment. Reliability estimates are along the diagonal in bold. All correlations |r| ≥ 0.13 are statistically significant with *p* < 0.05.

Next, we conducted a series of CFAs, with two factors representing precrastination and procrastination. The model loading all items onto one factor (CFI = 0.55; TLI = 0.52; SRMR = 0.153; RMSEA = 0.165) exhibited a significantly worse fit compared to the two-factor model with precrastination as factor one and procrastination as factor two (CFI = 0.74; TLI = 0.73; SRMR = 0.096; RMSEA = 0.125). We also ran an alternative model with three dimensions for precrastination and two dimensions for procrastination (given the two different measures we used). This model was the best-fitting model (CFI = 0.93; TLI = 0.91; SRMR = 0.049; RMSEA = 0.073). All item-level results for the five-factor CFA are reported in Table 3. Taken together, these results provide robust support for Hypothesis 1.

**Table 3:** Study 2 (Step 1): CFA factor loadings of workplace precrastination and procrastination items.

Item	CFA factor loadings				
	Precrastination – immediate start	Precrastination – rapid progress	Precrastination– early completion	Irrational procrastination scale (IPS)	Pure procrastination scale (PPS)
*As soon as I am given a task …*					
1. I quickly jump on it to finish it.	0.746				
2. I rush to start working on it.	0.775				
3. I begin working on it immediately.	0.940				
4. I start working on it right away.	0.935				
5. I act upon it immediately.	0.922				
6. I quickly start working on it.	0.922				
7. I work at a rapid pace.		0.847			
8. I expedite my working speed.		0.890			
9. I work rapidly to complete it.		0.944			
10. I move quickly to complete it.		0.915			
11. I make fast progress on it.		0.842			
12. I work fast to complete it.		0.914			
13. I complete my task with a lot of time to spare.			0.860		
14. I finish my task sooner than necessary.			0.871		
15. I finish my task long before the deadline.			0.861		
16. I complete my task earlier than required.			0.905		
17. I complete my task far ahead of the deadline.			0.863		
18. I complete my task earlier than needed.			0.909		
19. I put things off so long that my well-being or efficiency unnecessarily suffers.				0.804	
20. If there is something important I should do, I get to it after attending to less important tasks.				0.666	
21. My life would be better if I did some activities or tasks earlier (more quickly).				0.662	
22. When I should be doing one thing, I will do another.				0.837	
23. At the end of the day, I know I could have spent the time better.				0.811	
24. I do not spend my time wisely.				0.801	
25. I delay tasks beyond what is reasonable.				0.879	
26. I procrastinate.				0.794	
27. I do not do anything when it needs to be done.				0.812	
28. I delay making decision until it’s too late.					0.781
29. Even after I make a decision, I delay acting upon it.					0.837
30. I waste a lot of time on trivial matters before getting to the final decisions					0.802
31. In preparation for some deadlines, I often waste time by doing other things.					0.866
32. Even jobs that require little else except sitting down and doing them, I find that they seldom get done for days.					0.846
33. I often find myself performing tasks that I had intended to do days before.					0.839
34. I am continually saying “I’ll do it tomorrow”.					0.863
35. I generally delay before starting on work I have to do.					0.837
36. I find myself running out of time.					0.810
37. I do not get things done on time.					0.834
38. I am not very good at meeting deadlines.					0.739
39. Putting things off until the last minute has cost me money in the past.					0.731

Standardized factor loadings are shown.

As hypothesized (H2), there was a significant positive correlation between precrastination and workaholism ( $\bar{r}$ = 0.26, *p* < 0.001). All dimensions of precrastination were positively correlated with workaholism (IS: $\bar{r}$ = 0.21, *p* < 0.001; RP: $\bar{r}$ = 0.25, *p* < 0.001; EC: $\bar{r}$ = 0.19, *p* < 0.001). These findings provide support for Hypothesis 2. Next, we examined the relationships between precrastination and related variables (H3 and H4). Supporting Hypothesis 3a and 3b, all dimensions and the precrastination construct were positively related to positive affect ( $\bar{r}$ = 0.48, *p* < 0.001; IS: $\bar{r}$ = 0.45, *p* < 0.001; RP: $\bar{r}$ = 0.36, *p* < 0.001; EC: $\bar{r}$ = 0.39, *p* < 0.001), and negatively related to negative affect ( $\bar{r}$ = − 0.34, *p* < 0.001; IS: $\bar{r}$ = − 0.29, *p* < 0.001; RP: $\bar{r}$ = − 0.23, *p* < 0.001; EC: $\bar{r}$ = − 0.32, *p* < 0.001). As predicted by Hypothesis 4, promotion focus regulation was positively related to precrastination and its dimensions ( $\bar{r}$ = 0.55, *p* < 0.001; IS: $\bar{r}$ = 0.51, *p* < 0.001; RP: $\bar{r}$ = 0.46, *p* < 0.001; EC: $\bar{r}$ = 0.37, *p* < 0.001), and prevention focus regulation was positively related to precrastination and its dimensions ( $\bar{r}$ = 0.28, *p* = 0.002; IS: $\bar{r}$ = 0.30, *p* < 0.001; RP: $\bar{r}$ = 0.20, *p* = 0.003; EC: $\bar{r}$ = 0.21, *p* = 0.002).

### 3.4 Antecedents and outcomes (step 2)

We further tested the proposed antecedents and outcomes for precrastination. Structural equation modeling (SEM) was used to test the hypothesized model, given its capacity to test all hypothesized relationships simultaneously and control for measurement errors (Millsap, 2002). Table 2 provides descriptive statistics and correlations among the main study variables.

Before testing our hypotheses, we conducted CFA to validate the distinctiveness of variables in our model across two separate time waves. The hypothesized measurement model (Model 1) with items loading to their respective factors was compared to three alternative models (see Table 4). Given the high correlation among the precrastination dimensions, one alternative model combined the three dimensions of precrastination into a single factor (Model 2). Another alternative model had a three-factor solution that separated antecedent variables, precrastination, and outcome variables (Model 3). Finally, we tested a combined model with all items loaded onto a single latent factor (Model 4). The results indicated that the hypothesized measurement model was the best-fitting model compared to the alternative models, as evidenced by the chi-square difference test, ΔCFI value, and RMSEA fit indices, which met the suggested cutoffs for good model fit (ΔCFI ≥ 0.002; RMSEA values close to 0.06; Meade et al., 2008; Hu and Bentler, 1999). The one-factor model, where all items were loaded onto one factor, fit the data significantly weaker (RMSEA = 0.112, CFI = 0.337, TLI = 0.325, Δ*χ*^2^ (5564) = 14917.41, ΔCFI = 0.501, *p* < 0.001) than the hypothesized measurement model (RMSEA = 0.056, CFI = 0.838, TLI = 0.832).

**Table 4:** Study 2 (Step 2): CFA model fit of variables in the structural modeling test.

Model	*χ^2^*	*df*	RMSEA	CFI	TLI	*Δχ^2^*	ΔCFI
Model 1: The measurement model with precrastination dimensions	10251.05^***^	5,459	0.056	0.838	0.832		
Model 2: The measurement model with precrastination composite	12330.03^***^	5,498	0.067	0.769	0.762	2078.98^***^	0.069
Model 3: Three-factor (antecedents-precrastination-outcomes) model	19931.08^***^	5,561	0.096	0.514	0.505	9680.03^***^	0.324
Model 4: One-factor (all combined) model	25168.46^***^	5,564	0.112	0.337	0.325	14917.41^***^	0.501

Variables included in the CFA are precrastination, conscientiousness, personal initiation, trait anxiety, task interdependence, task autonomy, procrastination, task performance, citizenship performance, job satisfaction, work engagement, and organizational commitment. ***p < 0.001.

Because the item-level model would have exceeded the recommended parameter to sample size ratio for estimation (1:5; Bentler and Chou, 1987), item parcels were employed as observed indicators in SEM. We created three parcels for each variable (except for citizenship performance, where the three original items were used) by sequentially averaging the items with the highest and lowest loadings (Rogers and Schmitt, 2004) as indicators of variables in our hypothesized model. Due to the multidimensional nature of workplace precrastination, we followed the internal consistency approach (Kishton and Widaman, 1994) to form parcels with items from the same dimension, which would maximize the internal consistency of the parcel. Considering the complicated procedures of our estimated model, parceling is recommended to reduce the probability of violation of non-normality for maximum likelihood estimation and has been shown to improve model fit without biasing parameter estimates (Williams and O’Boyle, 2008).

We used SEM for hypothesis testing. In all, we computed five models (see Table 5). In Model I, we tested the hypothesized model with procrastination as a control variable. We computed four alternative models for robustness testing. We tested Model II with the three dimensions of precrastination as parallel mediators in the model. Model III examined the three dimensions as serial mediators, with the immediate start dimension preceding the other two dimensions. We further conducted two robustness checks, with Model IV testing the hypothesized model after removing insignificant paths, and Model V introducing direct paths from variables that yielded insignificant results to the outcomes. Fit statistics for the hypothesized model and all alternative models are included in Table 5. Because Model I represented our hypotheses with procrastination as a control and received a good model fit (RMSEA = 0.055, CFI = 0.95, TLI = 0.94, *χ*^2^ (776) = 1428.33, *p* < 0.001), we used parameter estimates from Model I for hypothesis testing.

**Table 5:** Study 2 (Step 2): fit statistics of structural modeling test.

Model	*χ^2^ (df)*	CFI	TLI	RMSEA	*Δχ^2^*	ΔCFI
Model I: Hypothesized model	1428.33^***^ (776)	0.95	0.94	0.055		
Model II: IS, RP, and EC dimensions as parallel mediators	1544.52^***^ (759)	0.94	0.93	0.061	116.19^***^	0.01
Model III: IS and RP and EC dimensions as serial mediators	1410.56^***^ (757)	0.95	0.94	0.056	17.77^***^	0.00
Model IV: Removing insignificant paths	1430.96^***^ (778)	0.95	0.94	0.055	2.63^***^	0.00
Model V: Introducing direct paths from insignificant antecedents to outcomes	1365.67^***^ (766)	0.95	0.95	0.053	62.66^***^	0.00

IS = immediate start; RP = rapid progress; EC = early completion. ***p < 0.001.

**Figure 1:**
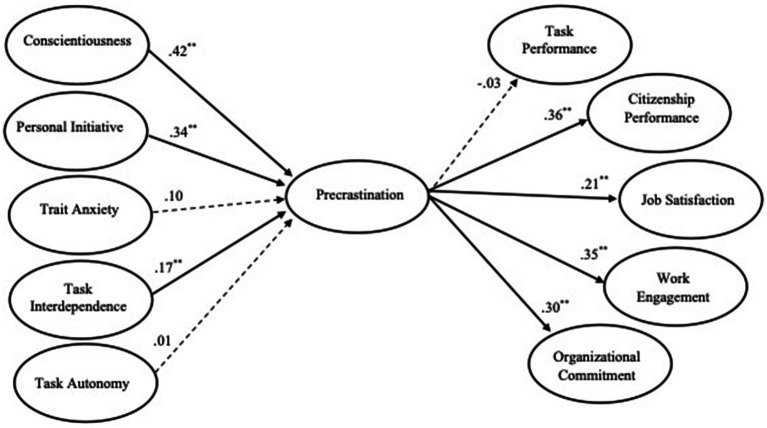
Study 2 (Step 2): hypothesized structural model estimates (Model I). CFI = 0.95, TLI = 0.94, RMSEA = 0.055. Standardized parameter estimates are shown, dotted lines represent hypothesized but nonsignificant paths. *p* < 0.01.

Figure 1 shows the standardized parameter estimates for each of the paths specified in Model I, with solid lines indicating significant paths and dotted lines hypothesized paths that were not significant. Hypothesis 5 predicted positive relationships between conscientiousness and precrastination, and the hypothesis was supported ( β = 0.42, *p* <0 .001). Hypothesis 6 was supported, with personal initiative positively related to precrastination ( β = 0.34, *p* =0 .003). Hypothesis 7 proposed a positive relationship between trait anxiety and precrastination, which was not supported ( β = 0.10, *p* =0 .103). Hypotheses 8a and 8b predicted positive relationships between task characteristics and precrastination. The relationship between task interdependence and precrastination ( β = 0.17, *p* =0 .002) was significant; however, the proposed relationship between task autonomy and precrastination ( β = 0.01, *p* =0 .851) was not supported. Hypotheses 9a and 9b predicted positive relationships between precrastination and performance outcomes. A significant relationship was observed between precrastination and citizenship performance ( β = 0.36, *p* <0 .001); however, the linkage with task performance ( β = − 0.03, p =0 .649) was not supported. For the attitudinal outcomes, Hypotheses 10a, 10b, and 10c were supported by the results, which indicates the presence of a positive relationship between precrastination and job satisfaction ( β = 0.21, *p* =0 .006), work engagement ( β = 0.35, *p* <0 .001), and organizational commitment ( β = 0.30, *p* <0 .001).

### 3.5 Incremental validity testing (step 3)

We conducted incremental validity analyses to determine whether our precrastination scale adds unique variance to the criterion variables above and beyond that explained by procrastination. We tested the incremental validity hypotheses with hierarchical multiple regression. Gender, age, and tenure were entered in Step 1, followed by procrastination in Step 2, and, finally, precrastination was entered in Step 3. We included demographic variables in our model because gender, age, and tenure are commonly suggested to influence performance and attitudes at work. Gender was coded as 1 for male and 2 for female. Age was measured in years, while tenure was assessed in months.

Given the high correlation among our precrastination dimensions ( $\bar{r}$ ranges from 0.55 to 0.68, *p* < 0.001), we adopted the approach outlined by LeBreton et al. (2007) to mitigate the potential issue of multicollinearity. Specifically, we employed relative weight analyses (RWAs) to determine the contribution of each precrastination dimension to the total explained variance (*R^2^*), thereby addressing concerns of variable collinearity through a variable transformation approach. This involved conducting RWAs with 95% bias-corrected and accelerated confidence intervals with 10,000 replications to assess the significance of the relative weights (Tonidandel and LeBreton, 2015). We also included rescaled relative weights to get the percentage of unique variance explained by each precrastination dimension. All analyses were conducted in R using the lavaan (Rosseel, 2012) and QuantPsyc (Fletcher, 2008) packages.

Table 6 presents the incremental validity results for all outcome variables. For performance variables, the effect of precrastination on task performance was not significant after controlling for procrastination, so Hypothesis 9a was not supported. According to the RWA results, although precrastination contributed only marginally to the increment in validity over procrastination (Δ*R^2^* = 0.01), its relative weight was in the expected direction (*RW* = 0.02) and it contributed to 10.75% of the total *R^2^* when compared to other predictors. Additional analyses after removing procrastination from the test suggested that precrastination contributed significant variance to task performance (*RW* = 0.04) and it contributed 73.63% of the total *R^2^*. This additional analysis suggested that precrastination predicts task performance, but its strength diminishes in the presence of procrastination. Aligned with our Hypothesis 9b, precrastination exhibited a significant positive effect on citizenship behavior ( β = 0.18; *p* < 0.001). As indicated by the change in *R^2^* values, the precrastination scale provided unique variance in the prediction of citizenship behavior (Δ*R^2^* = 0.04; *p* < 0.001). RWA revealed a similar pattern, with precrastination accounting for a larger proportion of the total predicted variance in citizenship behavior (47.25% of the total *R^2^*) compared to all other predictors (46.40% of the total *R^2^*).

**Table 6:** Study 2 (Step 3): incremental validity tests using precrastination composite scores.

Predictor	Raw important estimates			Rescaled estimates	Incremental importance
	*β*	RW	[RW CI]	RS-RW (%)	Δ*R^2^*
Criterion: task performance (*R*^2^ = 0.14)					
Gender	−0.03	0.00	[0.00, 0.02]	1.56	0.00
Age	0.00	0.01	[0.00, 0.03]	4.73	0.02
Tenure	0.00	0.00	[0.00, 0.01]	1.71	0.00
Procrastination	−0.18^***^	0.11	[0.05, 0.19]	81.24	0.12
Totals		0.12		89.23	0.14^***^
Precrastination	−0.02	0.02	[0.01, 0.06]	10.75	0.01
Criterion: Citizenship performance (*R*^2^ = 0.16)					
Gender	−0.15^*^	0.02	[0.00, 0.07]	13.83	0.03^**^
Age	0.00	0.01	[0.00, 0.05]	8.39	0.02^**^
Tenure	0.01	0.01	[0.00, 0.04]	7.23	0.01
Procrastination	−0.06	0.03	[0.00, 0.07]	16.95	0.06^***^
Totals		0.19		46.40	0.12
Precrastination	0.18^***^	0.08	[0.03, 0.14]	47.25	0.04^***^
Criterion: Job satisfaction (*R*^2^ = 0.12)					
Gender	0.02	0.00	[0.00, 0.00]	0.09	0.00
Age	0.00	0.00	[0.00, 0.01]	1.96	0.01
Tenure	0.00	0.00	[0.00, 0.02]	2.12	0.00
Procrastination	−0.22^**^	0.06	[0.02, 0.12]	50.14	0.09^***^
Totals		0.06		54.31	0.10^***^
Precrastination	0.24^**^	0.05	[0.02, 0.11]	45.69	0.02^**^
Criterion: Work engagement (*R*^2^ = 0.19)					
Gender	0.09	0.00	[0.00, 0.01]	0.67	0.00
Age	0.01	0.01	[0.00, 0.03]	4.20	0.02^*^
Tenure	0.01	0.01	[0.00, 0.03]	2.65	0.00
Procrastination	−0.13	0.06	[0.02, 0.12]	30.84	0.10^***^
Totals		0.08		38.36	0.12
Precrastination	0.37^***^	0.12	[0.06, 0.18]	61.63	0.07^***^
Criterion: Organizational commitment (*R*^2^ = 0.11)					
Gender	−0.07	0.00	[0.00, 0.02]	2.58	0.00
Age	0.01	0.01	[0.00, 0.04]	9.52	0.02^*^
Tenure	0.01	0.01	[0.00, 0.04]	10.47	0.01
Procrastination	−0.03	0.02	[0.00, 0.05]	18.06	0.03^**^
Totals		0.04		40.63	0.06^**^
Precrastination	0.33^***^	0.06	[0.02, 0.12]	59.38	0.05^***^

Sample 3 (Wave 1) = 291. Sample 3 (Wave 2) = 286. RW = raw relative weight (raw weights will sum to *R*^2^); [RW CI] = 95% confidence interval of raw weight; RS – RW (%) = relative weight rescaled as a percentage of predicted variance attributable to each predictor. ^a^Coding: 1, male; 2, female. ^b^Discrepancies in total are due to rounding. *p < 0.05; **p < 0.01; ***p < 0.001.

Turning next to the attitude variables (H10a, H10b, and H10c), precrastination showed significant positive effects on job satisfaction ( β = 0.24; *p* = 0.01), work engagement ( β = 0.37; *p* < 0.001), and organizational commitment ( β = 0.33; *p* < 0.001). Specifically, the precrastination scale contributed unique variance to the prediction of job satisfaction (Δ*R^2^* = 0.02; *p* = 0.01). Similarly, the RWA revealed that precrastination accounted for a large proportion of the total predicted variance in job satisfaction (*RW* = 0.05; 45.69% of the total *R^2^*). Thus, Hypothesis 10a was supported. For work engagement, precrastination contributed unique variance to the total prediction (Δ*R^2^* = 0.07; *p* < 0.001). Aligned with these results, the RWA showed a relative weight for precrastination (61.63% of the total *R^2^*) that was more than two times larger than that of procrastination (30.84% of the total *R^2^*). Thus, Hypothesis 10b was supported. Furthermore, precrastination added unique variance to the prediction of organizational commitment (Δ*R^2^* = 0.05; *p* < 0.001). These results were consistent with the RWA, where the total relative weights of precrastination (59.38% of the total *R^2^*) were larger than those of other predictors (40.63% of the total *R^2^*). Thus, Hypothesis 10c was supported.

### 3.6 Supplementary analysis

Given the high intercorrelations among the three dimensions of the workplace precrastination scale, we conducted two sets of supplementary analyses to provide additional evidence for the three-factor model and non-redundancy among the dimensions. First, built on our discriminant validity results in section 2.4 (Step 4), an alternative a second-order CFA was estimated, with a general precrastination factor loading on IS, RP, and EC. This model improved global fit relative to the first-order three-factor model (CFI/TLI increased from 0.94/0.93 to 0.96/0.95; RMSEA decreased from 0.10 to 0.07; AIC/BIC lower). Specifically, the higher-order factor explained 48–78% of variance in the three facets, indicating a coherent general precrastination factor. Moreover, facet-specific variance remained (IS = 34%; RP = 22%; EC = 52%), indicating distinctiveness among facets.

Second, we tested the incremental validity hypotheses using dimensional scores of workplace precrastination. The three dimensions showed meaningful predictive power over procrastination on performance and attitudinal outcomes. For example, the IS dimension provided unique variance in the prediction of citizenship behavior (Δ*R^2^* = 0.03; *p* < 0.01), job satisfaction (Δ*R^2^* = 0.02; *p* < 0.01), and work engagement (Δ*R^2^* = 0.04; *p* < 0.001). Moreover, the FE dimension explained the largest proportion of the total predicted variance in citizenship behavior (21.80% of the total *R^2^*) and organizational commitment (29.21% of the total *R^2^*). These results offered additional evidence that the three dimensions are distinct and contain unique predictive value in performance and attitude variables.^2^

## 4 Discussion

The concept of precrastination has largely been overlooked in applied psychology and organizational studies. In this study, we reviewed and integrated the extant literature and offered a new definition of workplace precrastination. Our conceptualization contributes to a deeper understanding of the nature and dimensions of this construct. Moreover, we developed and validated a psychometrically sound measure of precrastination. We also demonstrated empirical evidence for the validity of the newly developed scale using two student samples and one two-wave working adult sample. We organize the discussion sections based on the three questions the current study focused on.

### 4.1 Theoretical implications

#### 4.1.1 What is precrastination?

To better understand the construct of workplace precrastination, the current study synthesized the existing precrastination literature (Blinch and DeWinne, 2019; Rosenbaum et al., 2014), as well as the more general literature on task sequencing preferences (Fournier et al., 2019) to identify the characteristics of workplace precrastination. We defined *workplace precrastination* as the behavioral tendency of an employee to initiate, progress, and complete official tasks well in advance of the organizational expectations or deadlines. With a clear, operationalizable definition of precrastination, we demonstrated several lines of evidence showing that the newly developed precrastination measure has psychometrically sound properties. The alpha coefficient reliability estimates were constantly high across multiple samples. The EFA results demonstrated a three-factor structure (Table 1), and the CFA results further confirmed that the three-factor model was the best-fitting model (Table 3), which suggests that workplace precrastination is a multidimensional construct, as we proposed.

#### 4.1.2 Are precrastination and procrastination two ends of the same continuum or distinct constructs?

We provide theoretical arguments and empirical evidence suggesting that precrastination and procrastination are distinct constructs. Theoretically, we illustrated that immediate start, rapid progress, and early completion are essential facets of workplace precrastination, distinguishing it from procrastination. While low procrastination refers to not postponing intended tasks (Chu and Choi, 2005), precrastination specifically involves actively completing the tasks early, often with urgency, which differs from the even distribution of work over time. The results of our studies supported this theorization.

Our results indicated negative correlations between the newly developed precrastination scale and two existing procrastination measures (−0.51 and −0.56), which suggests that the precrastination measure is related to, but not redundant with, the existing measures of procrastination. Additionally, CFAs demonstrated that a two-factor model (precrastination and procrastination) provided a better model fit than a one-factor model. Further analyses with a five-factor model (the three dimensions of precrastination and two procrastination measures) provided the best fit, thus supporting the validity of the proposed multidimensional nature of precrastination (Table 3). In addition, the results of the hierarchical regression indicated that precrastination explained incremental variance in predicting organizational outcomes above and beyond procrastination (Table 6; Appendix Table 7), which suggests that precrastination has unique characteristics and predictive power.

#### 4.1.3 How is precrastination associated with important organizational constructs?

We provide evidence to establish the initial nomological network of precrastination. The results indicated that precrastination has positive relationships with workaholism, positive affect, and both promotion and prevention regulatory focus but has a negative relationship with negative affect. We further investigated the potential antecedents (e.g., conscientiousness, personal initiation, trait anxiety, and task characteristics) and consequences (e.g., task performance, citizenship performance, job satisfaction, work engagement, and organizational commitment) of precrastination (Figure 1). In line with the hypotheses, employee conscientiousness, personal initiative, and task interdependence were positively associated with precrastination. Precrastination was also associated with work attitudes (job satisfaction, work engagement, and organizational commitment) and citizenship performance (Table 6).

### 4.2 Practical implications

Our research provides managers insights regarding the benefits of precrastination in the workplace, thus empowering them to devise strategies to promote precrastination and enhance efficiency. Given the positive associations between precrastination and beneficial behaviors (e.g., personal initiative, self-regulatory behaviors) and outcomes (job satisfaction, work engagement, and organizational commitment) found in this study, managers could create a supportive environment that facilitates precrastination by clearly outlining task deadlines, providing details about the tasks, and, when possible, allowing uninterrupted time for employees to execute their plans. Managers could also intentionally encourage precrastination in employees by promptly responding to their emails, emphasizing the importance of specific projects, or reminding them of project workloads and due dates. Furthermore, given our results showing that precrastinators tend to exhibit higher citizenship behaviors, organizations should focus on hiring employees who are likely to complete their tasks early, because they are also likely to assist their colleagues and produce better outcomes for their teams.

Moreover, given the relationship between task interdependence and precrastination in this study, employees who start tasks quickly and complete them early can be valuable in the interdependent team settings where collaboration demand is high. Managers can leverage this precrastination tendency by assigning precrastinators to tasks with tight deadlines or roles that require early preparation and support for teammates.

Despite possible advantages of precrastination, excessive precrastination may carry some risks. For instance, finishing work too early may lead to rework, errors, or inaccuracy if important information emerges later in the work process. To avoid these downsides, managers should balance employees’ early-start tendencies with checkpoints, reflection periods, and clear guidance about when early completion is beneficial versus when waiting is wiser. Taken together, organizations should leverage precrastinators’ responsiveness while actively managing the risks associated with excessive haste.

### 4.3 Limitations and future directions

As with all research, it is important to note the limitations of this study. One limitation is the reliance on self-reported data. While this might introduce common method bias (Podsakoff et al., 2013), we sought to mitigate this bias wherever possible. For the incremental validity tests, data were collected at different time points to separate the measurement of the predictor and outcome variables. In the remaining validity tests, common bias may be less of concern, as most variables measured perceptions and attitudes, which are commonly assessed through self-reported data (Markoczy, 1997). Nonetheless, future researchers should obtain data from alternative sources (e.g., supervisor evaluations of subordinate performance) for certain outcome variables (e.g., task performance, citizenship performance) to further mitigate concerns related to common method bias.

The second concern is related to the relationship between precrastination and task performance. Although workplace precrastination correlated positively with task performance at the zero-order ( $\bar{r}$ = 0.18, *p* < 0.05; IS: $\bar{r}$ = 0.15, *p* < 0.05; RP: $\bar{r}$ = 0.11, n.s.; EC: $\bar{r}$ = 0.19, *p* < 0.05), its relationship became non-significant once procrastination was included in the model. Relative-weights analyses indicated that precrastination accounted for unique variance in task performance when procrastination was omitted (RW = 0.04; 73.63% of total R^2^). These results suggest that precrastination and procrastination may affect performance in somewhat overlapping ways, and the unique contribution of precrastination to performance is smaller than it first appears. These results should also be interpreted with caution, given that task performance was self-reported. Thus, future research should use objective or other-rated performance measures. Furthermore, future research may consider exploring other outcomes that might be more directly tied to the timing and quality of work, such as creativity, decision-making quality, long-term success. For example, creativity often benefits from slower, more reflective processes (Shin and Grant, 2021), making it a particularly interesting area for studying precrastination.

Third, in SEM, we used parcels to avoid over-parameterization relative to sample size (Bentler and Chou, 1987) and to mitigate violations of non-normality in maximum likelihood estimation (Williams and O’Boyle, 2008). Nonetheless, this approach should be interpreted with caution as parceling can also hide meaningful differences between items and even potentially produce a favorable model fit. Accordingly, future work should test our models using item-level data when a sufficiently large sample size is available and compare results across different ways of grouping items to ensure more robust conclusions.

Fourth, although our results present a preliminary nomological network of precrastination, our model may have overlooked other variables that could enhance this network. For example, completing a task way ahead of a deadline could sometimes result in errors and lower-quality output. Further research could therefore explore whether and how precrastination might be linked to negative outcomes. In addition, future research should extend our work by including a broader range of variables that could expand our understanding of mechanisms through which precrastination is associated with other psychological and organizational outcomes.

Finally, because all samples in this study were collected in the United States (i.e., university students and adult workers recruited via Prolific), the generalizability of our findings might be limited. For example, although our adult sample included online participants from multiple industries, which provides some occupational heterogeneity, future research should seek to replicate these findings with organizational field samples and cross-cultural populations. Prior work suggests that precrastination may vary across cultural and social settings (Rosenbaum et al., 2019), which underscores the need for cross-cultural validation of this scale.

### 4.4 Conclusion

Precrastination is prevalent in everyday life, yet it has received little attention and lack of a proper measure of precrastination in organizational research. To address this gap, we propose and develop a psychologically appropriate measure of precrastination. Our findings provide empirical evidence that precrastination is a distinct, multifaceted construct that differs from procrastination. By offering a validated measure that captures its key dimensions (immediate start, rapid progress, and early completion), this study provides researchers and practitioners with a valuable tool to understand the role of precrastination in the workplace. We hope that this newly developed precrastination measure will stimulate further scientific inquiry and broaden understanding of this timely and important phenomenon.

## Data Availability

The datasets presented in this study can be found in online repositories. The names of the repository/repositories and accession number(s) can be found at: in OSF Storage at: https://osf.io/he8ys/?view_only=a29d3b1b59f040b6bd809be88db89559.

## Footnotes

^1^To empirically address these conceptual limitations, we conducted a separate study to examine the factor structure of the precrastination scale by Gehrig et al. (2023), and the results show multidimensionality with some unclear patterns, suggesting that some items are confounded with something else. The detailed results of this study are shown in the Appendix A.

^2^Given the page limit, we have reported a summary of the results, but full results are available upon request.

## References

[ref1] Ancona D. G. Okhuysen G. A. Perlow L. A. (2001). Taking time to integrate temporal research. Acad. Manag. Rev. 26, 512–529. doi: 10.5465/amr.2001.5393887

[ref2] Batt R. J. Terwiesch C. (2017). Early task initiation and other load-adaptive mechanisms in the emergency department. Manag. Sci. 63, 3531–3551. doi: 10.1287/mnsc.2016.2516, PMID: 19642375

[ref9009] Bennett R. J. Robinson S. L. (2000). Development of a measure of workplace deviance. Journal of Applied Psychology, 85, 349–360. doi: 10.1037/0021-9010.85.3.349 10900810

[ref3] Bentler P. M. Chou C. P. (1987). Practical issues in structural modeling. Sociol. Methods Res. 16, 78–117. doi: 10.1177/0049124187016001004

[ref9010] Berkowitz L. Donnerstein E. (1982). External validity is more than skin deep: Some answers to criticisms of laboratory experiments. American Psychologist, 37, 245–257. doi: 10.1037/0003-066X.37.3.245

[ref4] Berry J. A. Tucker A. L. (2017). Past the point of speeding up: the negative effects of workload saturation on efficiency and patient severity. Manag. Sci. 63, 1042–1062. doi: 10.1287/mnsc.2015.2387

[ref5] Bledow R. Schmitt A. Frese M. Kühnel J. (2011). The affective shift model of work engagement. J. Appl. Psychol. 96, 1246–1257. doi: 10.1037/a0024532, PMID: 21766997

[ref6] Blinch J. DeWinne C. R. (2019). Pre-crastination and procrastination effects occur in a reach-to-grasp task. Exp. Brain Res. 237, 1129–1139. doi: 10.1007/s00221-019-05493-3, PMID: 30783715

[ref7] Blount S. Janicik G. A. (2001). When plans change: examining how people evaluate timing changes in work organizations. Acad. Manag. Rev. 26, 566–585. doi: 10.5465/amr.2001.5393892

[ref8] Borman W. C. Motowidlo S. J. (1997). Task performance and contextual performance: the meaning for personnel selection research. Hum. Perform. 10, 99–109. doi: 10.1207/s15327043hup1002_3

[ref10] Cheng B. H. McCarthy J. M. (2018). Understanding the dark and bright sides of anxiety: a theory of workplace anxiety. J. Appl. Psychol. 103, 537–560. doi: 10.1037/apl0000266, PMID: 29355338

[ref11] Chu A. H. Choi J. N. (2005). Rethinking procrastination: positive effects of “active” procrastination behavior on attitudes and performance. J. Soc. Psychol. 145, 245–264. doi: 10.3200/SOCP.145.3.245-264, PMID: 15959999

[ref12] Clark M. A. Smith R. W. Haynes N. J. (2020). The multidimensional workaholism scale: linking the conceptualization and measurement of workaholism. J. Appl. Psychol. 105, 1281–1307. doi: 10.1037/apl0000484, PMID: 32039607

[ref13] Costa P. T. McCrae R. R. (1992). Revised NEO personality inventory (NEO PI-RTM) and NEO five-factor inventory (NEO-FFI): Professional manual. Florida: Psychological Assessment Resources.

[ref14] Dalal R. S. Lam H. Weiss H. M. Welch E. R. Hulin C. L. (2009). A within-person approach to work behavior and performance: concurrent and lagged citizenship-counterproductivity associations, and dynamic relationships with affect and overall job performance. Acad. Manag. J. 52, 1051–1066. doi: 10.5465/amj.2009.44636148

[ref9006] DeMelo J. (2019). Precrastination: When the early bird gets the shaft. The New York Times. Available online at: https://www.nytimes.com/2019/03/25/smarter-living/precrastination-when-the-early-bird-gets-the-shaft.html

[ref9007] DeShon R. P. (2002). Generalizability theory. In Measuring and analyzing behavior in organizations: Advances in measurement and data analysis (pp. 189–220). Jossey-Bass.

[ref9008] Elliot A. J. McGregor H. A. (1999). Test anxiety and the hierarchical model of approach and avoidance achievement motivation. Journal of Personality and Social Psychology, 76, 628–644. doi: 10.1037/0022-3514.76.4.628 10234849

[ref15] Fletcher T. D. (2008). *QuantPsyc: Quantitative psychology tools*. R package Version 1.5 [Computer software]. Available online at: https://cran.r-project.org/web/packages/QuantPsyc/index.html.

[ref16] Fournier L. R. Coder E. Kogan C. Raghunath N. Taddese E. Rosenbaum D. A. (2019). Which task will we choose first? Precrastination and cognitive load in task ordering. Atten. Percept. Psychophys. 81, 489–503. doi: 10.3758/s13414-018-1633-5, PMID: 30506327

[ref17] Frese M. Fay D. Hilburger T. Leng K. Tag A. (1997). The concept of personal initiative: operationalization, reliability and validity in two German samples. J. Occup. Organ. Psychol. 70, 139–161. doi: 10.1111/j.2044-8325.1997.tb00639.x

[ref18] Gehrig C. Münscher J. C. Herzberg P. Y. (2023). How do we deal with our daily tasks? Precrastination and its relationship to personality and other constructs. Personal. Individ. Differ. 201, 111927–111924. doi: 10.1016/j.paid.2022.111927, PMID: 40972618

[ref19] George J. M. Jones G. R. (2000). The role of time in theory and theory building. J. Manage. 26, 657–684. doi: 10.1177/014920630002600404

[ref20] Hackman J. R. Oldham G. R. (1975). Development of the job diagnostic survey. J. Appl. Psychol. 60, 159–170. doi: 10.1037/h0076546

[ref21] Higgins E. T. (1997). Beyond pleasure and pain. Am. Psychol. 52, 1280–1300. doi: 10.1037/0003-066X.52.12.1280, PMID: 9414606

[ref22] Hinkin T. R. (1998). A brief tutorial on the development of measures for use in survey questionnaires. Organ. Res. Methods 1, 104–121. doi: 10.1177/109442819800100106

[ref23] Hong Y. Liao H. Raub S. Han J. H. (2016). What it takes to get proactive: an integrative multilevel model of the antecedents of personal initiative. J. Appl. Psychol. 101, 687–701. doi: 10.1037/apl0000064, PMID: 26653528

[ref9005] Hu L. T. Bentler P. M. (1999). Cutoff criteria for fit indexes in covariance structure analysis: Conventional criteria versus new alternatives. Structural Equation Modeling: A Multidisciplinary Journal, 6, 1–55. doi: 10.1080/10705519909540118

[ref24] Indeed. (2024). *How to deal with unreasonable demands at work (with steps)*. Available online at: https://www.indeed.com/career-advice/career-development/how-to-deal-with-unreasonable-demands-at-work (Accessed November 24, 2024).

[ref9004] Izard C. E. Youngstrom E. A. (1996). The activation and regulation of fear and anxiety. In Hope DA (Ed.), Nebraska Symposium of Motivation. Vol. 43. Perspectives on anxiety, panic and fear (pp. 1–59). Lincoln: University of Nebraska Press. 8912307

[ref25] Judge T. A. Martocchio J. J. Thoresen C. J. (1997). Five-factor model of personality and employee absence. J. Appl. Psychol. 82, 745–755. doi: 10.1037/0021-9010.82.5.745

[ref26] Kishton J. M. Widaman K. F. (1994). Unidimensional versus domain representative parceling of questionnaire items: an empirical example. Educ. Psychol. Meas. 54, 757–765. doi: 10.1177/0013164494054003022

[ref27] Klein H. J. Cooper J. T. Molloy J. C. Swanson J. A. (2014). The assessment of commitment: advantages of a unidimensional, target-free approach. J. Appl. Psychol. 99, 222–238. doi: 10.1037/a0034751, PMID: 24188389

[ref28] Kline T. J. B. (2005). Psychological testing: A practical approach to design and evaluation. Newcastle upon Tyne: Sage.

[ref29] Kruglanski A. W. (1989). The psychology of being “right”: the problem of accuracy in social perception and cognition. Psychol. Bull. 106, 395–409. doi: 10.1037/0033-2909.106.3.395

[ref30] LeBreton J. M. Hargis M. B. Griepentrog B. Oswald F. L. Ployhart R. E. (2007). A multidimensional approach for evaluating variables in organizational research and practice. Pers. Psychol. 60, 475–498. doi: 10.1111/j.1744-6570.2007.00080.x

[ref31] Legood A. Lee A. Schwarz G. Newman A. (2018). From self-defeating to other defeating: examining the effects of leader procrastination on follower work outcomes. J. Occup. Organ. Psychol. 91, 430–439. doi: 10.1111/joop.12205, PMID: 30333685 PMC6175130

[ref32] Markoczy L. (1997). Measuring beliefs: accept no substitutes. Acad. Manag. J. 40, 1228–1242. doi: 10.5465/256934

[ref34] Meade A. W. Johnson E. C. Braddy P. W. (2008). Power and sensitivity of alternative fit indices in tests of measurement invariance. J. Appl. Psychol. 93, 568–592. doi: 10.1037/0021-9010.93.3.568, PMID: 18457487

[ref35] Meyer J. P. Herscovitch L. (2001). Commitment in the workplace: toward a general model. Hum. Resour. Manag. Rev. 11, 299–326. doi: 10.1016/S1053-4822(00)00053-X

[ref36] Millsap R. E. (2002). “Structural equation modeling: a user’s guide” in Measuring and analyzing behavior in organizations: Advances in measurement and data analysis. eds. Drasgow F. Schmitt N. (Hoboken, NJ: Jossey-Bass/Wiley), 257–301.

[ref37] Mitchell G. (2012). Revisiting truth or triviality: the external validity of research in the psychological laboratory. Perspect. Psychol. Sci. 7, 109–117. doi: 10.1177/1745691611432343, PMID: 26168439

[ref38] Mohammed S. Nadkarni S. (2011). Temporal diversity and team performance: the moderating role of team temporal leadership. Acad. Manag. J. 54, 489–508. doi: 10.5465/amj.2011.61967991

[ref39] Moon H. (2001). Looking forward and looking back: integrating completion and sunk-cost effects within an escalation-of-commitment progress decision. J. Appl. Psychol. 86, 104–113. doi: 10.1037/0021-9010.86.1.104, PMID: 11302222

[ref40] Morgeson F. P. Humphrey S. E. (2006). The work design questionnaire (WDQ): developing and validating a comprehensive measure for assessing job design and the nature of work. J. Appl. Psychol. 91, 1321–1339. doi: 10.1037/0021-9010.91.6.1321, PMID: 17100487

[ref41] Ng T. W. H. Sorensen K. L. Feldman D. C. (2007). Dimensions, antecedents, and consequences of workaholism: a conceptual integration and extension. J. Organ. Behav. 28, 111–136. doi: 10.1002/job.424

[ref9002] O’Donoghue T. Rabin M. (1999). Doing it now or later. American Economic Review, 89, 103–124. doi: 10.1257/aer.89.1.103

[ref42] Patterson E. E. Kahan T. A. (2020). Precrastination and the cognitive-load-reduction (CLEAR) hypothesis. Memory (Hove, England) 28, 107–111. doi: 10.1080/09658211.2019.1690001, PMID: 31726943

[ref43] Podsakoff P. M. MacKenzie S. B. Podsakoff N. P. (2016). Recommendations for creating better concept definitions in the organizational, behavioral, and social sciences. Organ. Res. Methods 19, 159–203. doi: 10.1177/1094428115624965

[ref44] Podsakoff N. P. Whiting S. W. Welsh D. T. Mai K. M. (2013). Surveying for “artifacts”: the susceptibility of the OCB-performance evaluation relationship to common rater, item, and measurement context effects. J. Appl. Psychol. 98, 863–874. doi: 10.1037/a0032588, PMID: 23565897

[ref8001] Richtel M. (2014). Sometimes, early birds are too early. New York Times. Available online at: https://www.nytimes.com/2014/07/20/business/sometimes-early-birds-are-too-early.html

[ref45] Rogers W. M. Schmitt N. (2004). Parameter recovery and model fit using multidimensional composites: a comparison of four empirical parceling algorithms. Multivar. Behav. Res. 39, 379–412. doi: 10.1207/S15327906MBR3903_1

[ref46] Rosenbaum D. A. (2020). *Now is not the time for precrastination*. APS Obs, No. 33. Available online at: https://www.psychologicalscience.org/observer/now-is-not-the-time-for-precrastination.

[ref47] Rosenbaum D. A. Fournier L. R. Levy-Tzedek S. McBride D. M. Rosenthal R. Sauerberger K.. (2019). Sooner rather than later: precrastination rather than procrastination. Curr. Dir. Psychol. Sci. 28, 229–233. doi: 10.1177/0963721419833652

[ref48] Rosenbaum D. A. Gong L. Potts C. A. (2014). Pre-crastination: hastening subgoal completion at the expense of extra physical effort. Psychol. Sci. 25, 1487–1496. doi: 10.1177/0956797614532657, PMID: 24815613

[ref49] Rosenbaum D. A. Sauerberger K. S. (2019). End-state comfort meets pre-crastination. Psychol. Res. 83, 205–215. doi: 10.1007/s00426-018-01142-6, PMID: 30623239

[ref50] Rosseel Y. (2012). Lavaan: an R package for structural equation modeling. J. Stat. Softw. 48, 1–36. doi: 10.18637/jss.v048.i02

[ref51] Sauerberger K. (2019). *When doing things later is the best choice: Precrastination as an individual difference [ProQuest Information & Learning]*. In Dissertation abstracts international: Section B: The sciences and engineering 4–B, p. 81.

[ref52] Sauerberger K. S. Rosenbaum D. A. Funder D. C. (2018). *When doing things later is the best choice: Precrastination as an individual difference*. Poster session presented at the 2018 annual meeting of the Society for Personality and Social Psychology, Atlanta, GA.

[ref53] Schaufeli W. B. Bakker A. B. Salanova M. (2006). The measurement of work engagement with a short questionnaire: a cross-national study. Educ. Psychol. Meas. 66, 701–716. doi: 10.1177/0013164405282471

[ref54] Shin J. Grant A. M. (2021). When putting work off pays off: the curvilinear relationship between procrastination and creativity. Acad. Manag. J. 64, 772–798. doi: 10.5465/amj.2018.1471

[ref55] Shipp A. J. Richardson H. A. (2021). The impact of temporal schemata: understanding when individuals entrain versus resist or create temporal structure. Acad. Manag. Rev. 46, 299–319. doi: 10.5465/amr.2017.0384

[ref56] Spitzer R. L. Kroenke K. Williams J. B. Löwe B. (2006). A brief measure for assessing generalized anxiety disorder: the GAD-7. Arch. Intern. Med. 166, 1092–1097. doi: 10.1001/archinte.166.10.1092, PMID: 16717171

[ref57] Steel P. (2007). The nature of procrastination: a meta-analytic and theoretical review of quintessential self-regulatory failure. Psychol. Bull. 133, 65–94. doi: 10.1037/0033-2909.133.1.65, PMID: 17201571

[ref58] Steel P. (2010). Arousal, avoidant and decisional procrastinators: do they exist? Pers. Individ. Differ. 48, 926–934. doi: 10.1016/j.paid.2010.02.025

[ref59] Svartdal F. Steel P. (2017). Irrational delay revisited: examining five procrastination scales in a global sample. Front. Psychol. 8:927. doi: 10.3389/fpsyg.2017.01927, PMID: 29163302 PMC5676095

[ref9003] Tepper B. J. (2007). Abusive supervision in work organizations: Review, synthesis, and research agenda. Journal of Management, 33, 261–289. doi: 10.1177/0149206307300812

[ref60] Tett R. P. Toich M. J. Ozkum S. B. (2021). Trait activation theory: a review of the literature and applications to five lines of personality dynamics research. Annu. Rev. Organ. Psychol. Organ. Behav. 8, 199–233. doi: 10.1146/annurev-orgpsych-012420-062228

[ref61] Tonidandel S. LeBreton J. M. (2015). RWA web: a free, comprehensive, web-based, and user-friendly tool for relative weight analyses. J. Bus. Psychol. 30, 207–216. doi: 10.1007/s10869-014-9351-z

[ref62] Villarreal S. R.. (2020). *Procrastination vs. Precrastination: What determines what we do? [PhD thesis]*. Illinois State University.

[ref63] VonderHaar R. L. McBride D. M. Rosenbaum D. A. (2019). Task order choices in cognitive and perceptual-motor tasks: the cognitive-load-reduction (CLEAR) hypothesis. Atten. Percept. Psychophys. 81, 2517–2525. doi: 10.3758/s13414-019-01754-z, PMID: 31073950

[ref64] Wallace C. Chen G. (2006). A multilevel integration of personality, climate, self-regulation, and performance. Pers. Psychol. 59, 529–557. doi: 10.1111/j.1744-6570.2006.00046.x

[ref65] Wasserman E. A. (2019). Precrastination: the fierce urgency of now. Learn. Behav. 47, 7–28. doi: 10.3758/s13420-018-0358-6, PMID: 30264372

[ref66] Watson D. Clark L. A. Tellegen A. (1988). Development and validation of brief measures of positive and negative affect: the PANAS scales. J. Pers. Soc. Psychol. 54, 1063–1070. doi: 10.1037/0022-3514.54.6.1063, PMID: 3397865

[ref67] Williams L. J. Anderson S. E. (1991). Job satisfaction and organizational commitment as predictors of organizational citizenship and in-role behaviors. J. Manage. 17, 601–617. doi: 10.1177/014920639101700305

[ref68] Williams L. J. O’Boyle E. H. (2008). Measurement models for linking latent variables and indicators: a review of human resource management research using parcels. Hum. Resour. Manag. Rev. 18, 233–242. doi: 10.1016/j.hrmr.2008.07.002

[ref69] Yam K. C. Fehr R. Barnes C. M. (2014). Morning employees are perceived as better employees: employees’ start times influence supervisor performance ratings. J. Appl. Psychol. 99, 1288–1299. doi: 10.1037/a0037109, PMID: 24911178

[ref9001] Yang F. Ding L. Lu M. Chen G. Bai C. Wang F. (2023). When boss puts off, the team worse off: The mitigating role of initiative-enhancing HRM systems. Group & Organization Management. Advance online publication. doi: 10.1177/10596011231223272

[ref70] Zermatten A. Van der Linden M. D'Acremont M. Jermann F. Bechara A. (2005). Impulsivity and decision making. J. Nerv. Ment. Dis. 193, 647–650. doi: 10.1097/01.nmd.0000180777.41295.65, PMID: 16208159

[ref71] Zhu M. Yang Y. Hsee C. K. (2018). The mere urgency effect. J. Consum. Res. 45, 673–690. doi: 10.1093/jcr/ucy008

