# Supplementary material for: Workplace precrastination: conceptualization and scale development
Source: Front Psychol. 2025 Oct 6;16:1679477. doi: 10.3389/fpsyg.2025.1679477 (PMC12535967; doi:10.3389/fpsyg.2025.1679477)
Supplement: Supplementary file 1 [file Table_1.docx]

**Appendix A**

To empirically test whether the scale developed by Gehrig et al. (2023) shows precrastination as a unidimensional construct, we collected data to explore the factor structure of their precrastination scale.

**1 Participants and Procedure**

We collected data from a sample of 153 undergraduate students majoring in management from a mid-sized southeast U.S. university. The mean age of participants was 21.99 years (*SD* = 3.76), with 58.17% identifying as male, and 88.24% identifying as White. About 75.16% of the respondents reported current employment, with 18.95% with full-time and an average of 4.58 years of experience in their current positions (*SD* = 3.63). In the survey, participants were asked to rate the extent to which they agree with the items in the 10-item precrastination measure from Gehrig et al. (2023) on a 5-point Likert-type scale, followed by a series of demographic questions.

**2 Results and Discussion**

An exploratory factor analysis (maximum likelihood with Promax rotation) was conducted to examine the underlying factor structure of Gehrig et al (2023)’s precrastination scale. The results showed a three-factor structure with minimum eigenvalue of .28 and a total variance explained of 60.97%. Unlike the originally proposed unidimensional structure in Gehrig et al. (2023), our findings showed a three-dimensional structure with unclear patterns, which illustrates potential limitations of this scale.

Specifically, seven items loaded onto the first factor, with one item, “It’s important to me to get my to-do list done on time,” had a relatively low factor loading of .50. The second factor contained two items, including “Starting a task early gives me a relieving feeling” with a factor loading at .57. As we predicted, the poor loading of this item validates concerns that it confounds expected emotions associated with precrastination with precrastinatory behavior. The third factor contained only one item with a factor loading of .915, “Starting a task early is more important to me than finishing it early.” We believe this item reflects an ideological belief rather than a precrastination tendency. The factor loadings for the three-factor model are reported in Table 7. In summary, based on our theoretical arguments and empirical evidence from this additional study using a different sample, we argue that the precrastination scale developed by Gehrig et al. (2023) may not fully capture the existing conceptualization of precrastination. Rather, the items appear to confound precrastination tendency with emotions and beliefs.

**Table 7**

*Additional Study: Exploratory Factor Loadings of Unidimensional Precrastination Scale by Gehrig et al. (2023)*

| Item | Factors | | |
| --- | --- | --- | --- |
|  | *Factor 1* | *Factor 2* | *Factor 3* |
|  | | | |
| 1. I prefer to start tasks immediately. | **.836** | .050 | −.057 |
| 2. I prefer to do things sooner rather than later. | **.796** | .066 | .009 |
| 3. When I get a task, I usually do it immediately. | **.788** | −.105 | .071 |
| 4. I prefer to start my work sooner rather than later. | **.784** | .015 | .071 |
| 5. I always act according to the maxim “What you can do today, don’t put off until tomorrow.” | **.684** | .093 | .218 |
| 6. I prefer to do things right away, even if doing them right away means extra work for me. | **.652** | .079 | .033 |
| 7. It’s important to me to get my to-do list done on time. | **.496** | .485 | −.323 |
| 8. I do unimportant things immediately so that I have more time for the important things. | −.156 | **.816** | −.032 |
| 9. Starting a task early gives me a relieving feeling. | .134 | **.568** | .243 |
| 10. I complete my task with a lot of time to spare. | .122 | .112 | **.915** |

*Note.* N = 153. Factor loadings above .490 are in bold.

**Appendix B**

As part of the deductive item generation process, we reviewed twelve existing scales: the General Procrastination scale (Lay, 1986), the Procrastination at Work scale (Metin et al., 2016), Decisional Procrastination scale (Mann, 1982), the volitional competence self-regulatory scale (Steel, 2002), the Worrying scale (Van Eerde, 2003), the Aitken Procrastination Inventory (Aitken, 1982), the Tuckman Procrastination scale (Tuckman, 1991), Active Procrastination (Choi & Moran, 2009), Jenkins Activity (Type A) survey (Jenkins et al., 1979), UPPS-P Impulsive Behavior scale (Lynam et al., 2007), the Self-control scale (Tangney et al., 2004), and the Precrastination scale (Gehrig et al., 2023).

**References**

Aitken, M. E. (1982). A personality profile of the college student procrastinator (Doctoral dissertation, University of Pittsburgh, 1982). *Dissertation Abstracts International*, 43: 722.

Choi, J. N., & Moran, S. V. (2009). Why not procrastinate? Development and validation of a new active procrastination scale. *The Journal of Social Psychology*, 149(2): 195-212. <https://doi.org/10.3200/SOCP.149.2.195-212>

Gehrig, C., Münscher, J. C., & Herzberg, P. Y. (2023). How do we deal with our daily tasks? Precrastination and its relationship to personality and other constructs. *Personality and Individual Differences*, 201: 1-4. <https://doi.org/10.1016/j.paid.2022.111927>

Jenkins, C. D., Zyzanski, S. J., and Rosenman, R. H. (1979). *Jenkins Activity Survey*, Psychological Corp., New York.

Lay, C. H. (1986). At last, my research article on procrastination. *Journal of Research in Personality*, 20(4): 474-495. <https://doi.org/10.1016/0092-6566(86)90127-3>

Lynam, D.R., Smith, G. T., Cyders, M. A., Fischer, S., & Whiteside, S. A. (2007). The UPPS-P: A multidimensional measure of risk for impulsive behavior. Unpublished technical report.

Mann, L. (1982). Decision making questionnaire. Unpublished manuscript, Flinders University of South Australia.

Metin, U. B., Taris, T. W., & Peeters, M. C. (2016). Measuring procrastination at work and its associated workplace aspects. *Personality and Individual Differences*, 101: 254-263. <https://doi.org/10.1016/j.paid.2016.06.006>

Steel, P. D. G. (2002). *The measurement and nature of procrastination* (Vol. 63, Issues 3-B, p. 1599). ProQuest Information & Learning.

Tangney, J. P., Baumeister, R. F., & Boone, A. L. (2004). High self-control predicts good adjustment, less pathology, better grades, and interpersonal success. *Journal of Personality*, 72(2): 271–324. https://doi.org/10.1111/j.0022-3506.2004.00263.x

Tuckman, B. W. (1991). The development and concurrent validity of the procrastination scale. *Educational and Psychological Measurement*, 51(2): 473-480. <https://doi.org/10.1177/0013164491512022>

Van Eerde, W. (2003). Procrastination at work and time management training. *The Journal of Psychology*, 137(5): 421-434. <https://doi.org/10.1080/00223980309600625>
